# CORRIGENDUM

**DOI:** 10.1002/rcr2.921

**Published:** 2022-02-24

The authors would like to draw the reader's attention to an error in the following article:

*Sekimoto, Y, Nagata, Y, Kato, M, Arai, Y, Fujimoto, Y, Takagi, H, et al. Idiopathic dendriform pulmonary ossification diagnosed by bronchoscopic lung cryobiopsy: A case report. Respirology Case Reports. 2021; 10:e0896*. https://doi.org/10.1002/rcr2.896

The selling of the name of author Takehiko Shukuya is incorrect and should be Takehito Shukuya.

The authors apologize for this error and any confusion it may have caused.

